# Functional Analysis of Phagocyte Activity in Whole Blood from HIV/Tuberculosis-Infected Individuals Using a Novel Flow Cytometry-Based Assay

**DOI:** 10.3389/fimmu.2017.01222

**Published:** 2017-09-28

**Authors:** Ankur Gupta-Wright, Dumizulu Tembo, Kondwani C. Jambo, Elizabeth Chimbayo, Leonard Mvaya, Shannon Caldwell, David G. Russell, Henry C. Mwandumba

**Affiliations:** ^1^College of Medicine, Malawi-Liverpool-Wellcome Trust Clinical Research Programme, Blantyre, Malawi; ^2^Clinical Research Department, London School of Hygiene and Tropical Medicine, London, United Kingdom; ^3^Department of Microbiology and Immunology, College of Veterinary Medicine, Cornell University, Ithaca, NY, United States; ^4^Department of Clinical Sciences, Liverpool School of Tropical Medicine, Liverpool, United Kingdom

**Keywords:** phagocytosis, zymosan, inflammation, monocytes, neutrophils, HIV, tuberculosis, whole blood assay

## Abstract

The accurate assessment of immune competence through *ex vivo* analysis is paramount to our understanding of those immune mechanisms that lead to protection or susceptibility against a broad range of human pathogens. We have developed a flow cytometry-based, whole blood phagocyte functional assay that utilizes the inflammatory inducer zymosan, coupled to OxyBURST-SE, a fluorescent reporter of phagosomal oxidase activity. The assay measures both phagocytic uptake and the superoxide burst in the phagocyte populations in whole blood. We utilized this assay to demonstrate impaired superoxide burst activity in the phagocytes of hospitalized HIV-positive patients with laboratory-confirmed tuberculosis. These data validate the use of the assay to assess the immune competence of patients in a clinical setting. The method is highly reproducible with minimal intraindividual variation and opens opportunities for the rapid assessment of cellular immune competence in peripheral blood in a disease setting.

## Introduction

Bacterial killing assays in whole blood are well established and allow *ex vivo* assessment of immune function in patients, particularly in the context of assessing response to vaccines or evaluating new bactericidal therapies (1–4). The main read out of these assays is microbial killing measured *via* culture and colony counting, or fluorescence if reporter strain organisms are used.

Potential problems of these microbiological killing assays include difficulties in standardizing the number of microbes and their multiplication rate. The tendency of the microbes to aggregate inconsistently during assays may also result in misrepresentation of the actual numbers of microbes measured at the end of the assay. In addition, there are other factors that can result in microbial loss that are not dependent on the host immune response or antimicrobial therapy (5). Finally, because the read out is simply bacterial survival, these assays lack the ability to differentiate mechanisms of killing and the relative contributions of the different phagocyte lineages present in the blood.

Phagocytosis is an important mechanism in the microbial killing pathway of phagocytes. Deficiencies in phagocyte function likely predispose individuals to acquire or succumb to infectious diseases. An extensive range of dynamic assays of phagosome function have been developed that are capable of providing a broad range of physiological readouts from the phagosome (6, 7). These assays have mostly utilized inert beads derivatized with different fluorescent reporters and focused on human alveolar macrophages or murine bone marrow-derived macrophages in culture (8–10). By removing cells from whole blood or their usual tissue fluid, we are unable to assess the potentially important influence of soluble proteins such as cytokines, chemokines, or antibodies on phagocytosis and phagosomal behavior. We therefore sought to develop an assay using a reporter particle more suitable for probing phagocyte biology in whole blood. The assay is designed to provide reproducible, unbiased, real-time analysis of phagosomal function of immune cells and potentially identify patients with impaired immune responses.

We utilized zymosan derivatized with the oxidation-sensitive fluorescent reporter, OxyBURST-SE, to quantify phagosomal oxidase activity in peripheral blood phagocytes *in situ*. Zymosan is a preparation of a cell wall glucan from *Saccharomyces cerevisiae* that has been used as a model microbial particle in immune assays for over half a century (11). Zymosan is highly mannosylated and linked to β-glucan, making it susceptible to phagocytosis by monocytes, polymorphonuclear leukocytes, and macrophages through various receptors, including C-type lectin receptors such as dectin-1 and mannose receptors (12, 13). Phagocytosis of zymosan can occur independent of opsonization, of which complement factor 3 (C3) predominates with immunoglobulin G (IgG) being of minor importance (14). Zymosan also stimulates an inflammatory cytokine response *via* toll-like receptors (TLR) 2 and 6, although activation of these receptors is not required for internalization by phagocytes (12). We had demonstrated previously how inert particles coupled to OxyBURST-SE can be used to quantify the superoxide bust of murine macrophages *in vitro* (15).

Superoxide burst is one of the key enzymatic activities involved in killing microbes during the process of phagocytosis. The generation of oxygen radicals *via* nicotinamide adenine dinucleotide phosphate (NADPH) oxidase leads to the production of noxious compounds such as hydrogen peroxide with potent antimicrobial activity (16, 17). Superoxide burst’s importance is clearly demonstrated by the greatly increased risk of bacterial, fungal, and mycobacterial infection in patient with chronic granulomatous disease due to mutations in NADPH oxidase (18). It has also been shown to be suppressed in individuals with HIV infection (19) and by *Mycobacterium tuberculosis* (TB) infection *in vitro* (20).

In this study, we report the application of this novel reporter platform to quantify the phagocytic and superoxide burst functions of phagocytes in whole blood obtained from individuals in a clinical setting. First, we detail the information generated by application of the assay in whole blood from healthy controls. We then present data showing the utility of this assay in demonstrating the perturbation of phagocyte function in the blood from HIV- and TB-coinfected patients in Malawi.

## Materials and Methods

### Study Population

Adult patients with HIV and tuberculosis coinfection (HIV-TB) were recruited as part of a sub-study examining immune responses in the Malawi arm of the rapid urine-based screening for TB to reduce AIDS-related mortality in hospitalized patients in Africa (STAMP) (21). Healthy HIV-negative adults with no evidence of active TB were also recruited as controls. 5 ml of blood was collected from both patients and controls in sodium heparin tubes. All samples were processed and analyzed by flow cytometry at the Malawi-Liverpool-Wellcome Trust Clinical Research Programme in Blantyre, Malawi within 2 h of blood draw. The study has been approved by the London School of Hygiene & Tropical Medicine Research Ethics Committee and the College of Medicine Research Ethics Committee, Malawi.

### Zymosan Reporter Particles

To quantify both phagocytic activity and the magnitude of the superoxide burst we utilized zymosan particles coupled to both a calibration fluorochrome (Alexa Fluor 405-SE, Invitrogen) and an oxidation-sensitive fluorescent reporter (OxyBURST^®^ Green H_2_DCFDA-SE, Invitrogen). Zymosan reporter particles were prepared by washing 6 mg of zymosan (Sigma-Aldrich) three times in 1× phosphate-buffered saline (PBS) by centrifugation at 10,000 rpm for 1 min. Particles were resuspended in 950 µl coupling buffer (0.1 M boric acid to pH 8.0 with NaOH) containing 10 µl of 25 mg/ml OxyBURST-SE/DMSO stock solution and 5 µl of 5 mg/ml Alexa Fluor 405-SE/DMSO solution. The particles were mixed well and incubated on a tube rocker in the dark for 1 h at room temperature and washed with 1 ml of coupling buffer. The 1 h coupling with OxyBURST-SE and calibration fluorochrome was repeated twice. Finally, particles were washed three times with PBS and stored in 1 ml of PBS containing 0.01% sodium azide in the dark at 4°C generating a final stock concentration of approximately 5 × 10^6^ particles/ml.

### Whole Blood Assay

Zymosan reporter particles were prepared for the whole blood assay by washing 50 µl of stock Zymosan particle suspensions three times with 1 ml of RPMI-1640 to remove sodium azide and resuspended in 250 µl RPMI-1640 to give a 1:6 dilution and a final concentration of approximately 8 × 10^5^ particles/ml.

Whole blood was diluted 1:1 with warm RPMI-1640. 20 µl of washed and diluted reporter particles (containing approximately 2 × 10^4^ particles) were added to 1 ml of diluted blood and incubated at 37°C with rocking to ensure particles and cells remain in suspension. Diluted blood without zymosan reporter particles was also processed in parallel as control. Phagocytosis of zymosan reporter particles and superoxide burst was assessed at 10, 30, 60, 90, and 180 min after the addition of reporter particles.

100 µl of diluted blood was harvested from the zymosan reporter and biological control tubes 10 min before each time point for cell surface staining (as phagocytosis continues during cell surface staining of live cells). Once harvested, the diluted blood was stained with appropriately titrated concentrations of antibodies (anti-CD45 PerCP 1:33, anti-CD66b APC 1:50, and anti-CD14 PE-Cy7 1:100; all from BioLegend) for 10 min. Biological activity was arrested, red blood cells were lysed, and leukocytes fixed by adding 3 ml of BD FACS lysing solution (BD Biosciences), containing formaldehyde and diethylene glycol, to each tube and incubating at room temperature for 10 min. The cells were washed once with 1× PBS by centrifugation at 500 *g* for 10 min then resuspended in 500 µl 1× PBS. Counting beads (Countbright, Life Technologies) were added per the manufacturer’s instruction before acquisition on a CyAn ADP flow cytometer (Beckman Coulter, USA). The phagocytosis assay was performed in triplicate on the whole blood samples from healthy, HIV-negative adults. Data were analyzed using FlowJo version 10 (Treestar, USA).

In addition to the zymosan reporter assay, for HIV/TB-coinfected patients, immunophenotyping of monocytes in fresh whole blood was undertaken to investigate the association between monocyte phenotype and phagocytosis. In brief, 100 µl of fresh whole blood was stained with anti-CD45 Pacific Orange (Invitrogen), anti-HLA-DR PE-Cy7, anti-CD14 PE, and anti-CD16 FITC (all from BioLegend) for 10 min. Red blood cells were lysed, and leukocytes fixed with BD FACS lysing solution, washed once with 1× PBS by centrifugation at 500 *g* for 10 min then resuspended in 300 µl PBS for flow cytometry acquisition.

### Electron Microscopy (EM)

In parallel, 2 ml of whole blood from a healthy HIV-negative control was incubated with approximately 8 × 10^4^ zymosan reporter particles to confirm the zymosan particles were internalized by whole blood phagocytes. White blood cells were harvested after 10, 60, and 180 min by centrifugation at 500 *g* for 10 min and carefully pipetting out the buffy coat layer in buffered glutaraldehyde fixative solution (2.5% glutaraldehyde in 0.1 M sodium cacodylate, 5 mM CaCl_2_, 5 mM MgCl_2_, 0.1 M sucrose, pH 7.2). The samples were processed and stained for EM as described previously (22).

### Calculations and Statistical Analysis

The proportion of cells that had phagocytosed reporter particles was calculated based on expression of calibration fluorochrome, and absolute cell numbers calculated using counting beads. An “activity index” of phagocytosis and superoxide burst was calculated by subtracting the median fluorescence intensity of the negative cells from the positive cells, and dividing this by two times the robust SD of the negative cells (23). This method accounted for variations in auto fluorescence between cells from different individuals.

Statistical analysis was performed using GraphPad Prism 7 (GraphPad Software, USA) and Stata 11 (StataCorp, USA). Peak activity index (AI) was calculated and mean AI was compared between groups. The AI at each time point was also used to calculate the area under the curve. Means were compared using paired *t*-tests and median using Wilcoxon rank-sum.

## Results/Discussion

### Zymosan Uptake by Whole Blood Phagocytes

**Figure 1:**
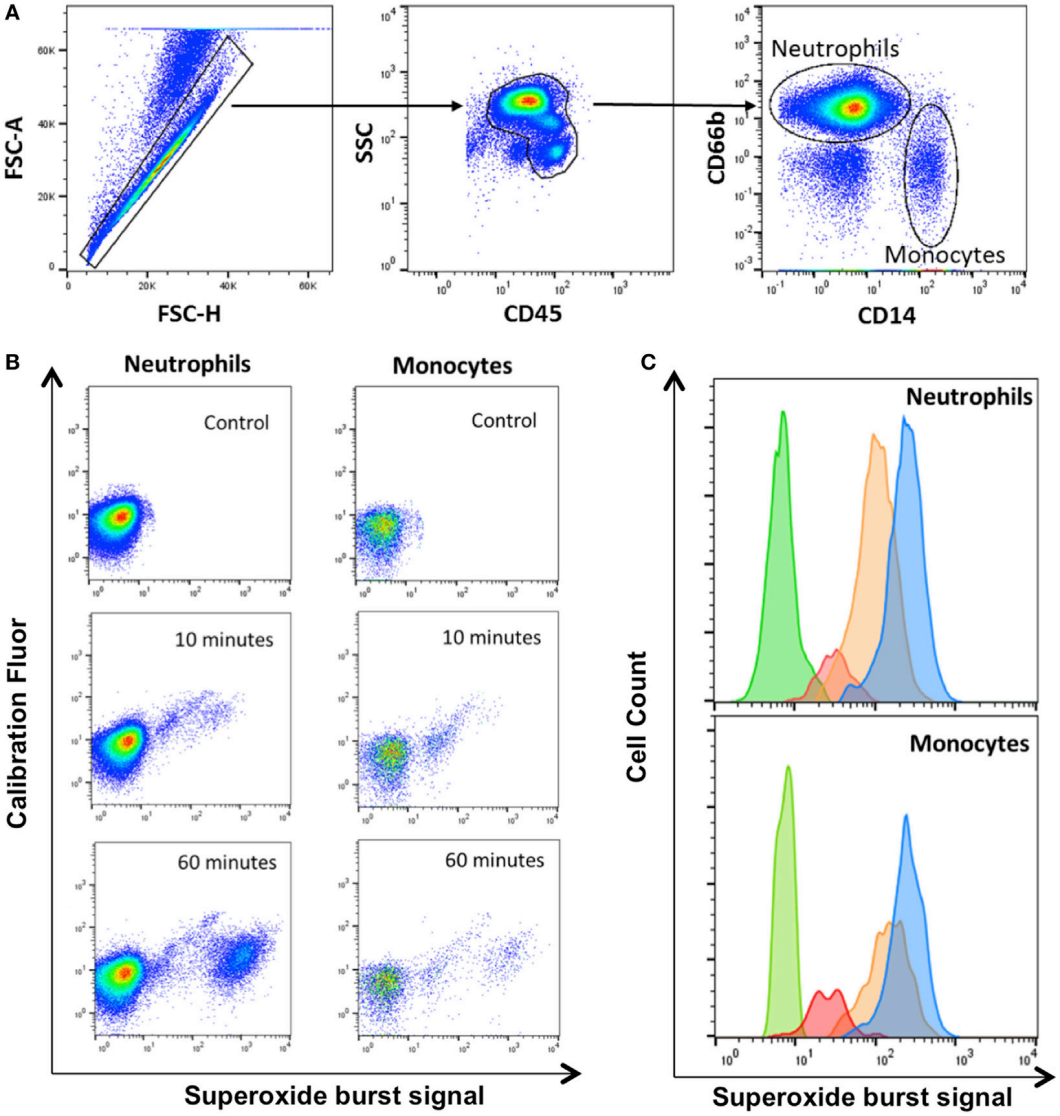
Gating strategy for identification of phagocytes with zymosan reporter particles and quantitation of intraphagosomal oxidation. **(A)** Gating strategy displaying forward scatter (FSC), side scatter (SSC), CD45 PerCP, CD66b APC, and CD14 PE-Cy7 to identify neutrophils and monocytes. The gating strategy illustrated is from one representative healthy volunteer. **(B)** Zymosan-induced superoxide burst activity in neutrophils and monocytes in a healthy control. The OxyBURST fluorescence increases after intraphagosomal oxidation of the zymosan-reporter particles (Alexa Fluor 405-labeled) after 10 and 60 min compared with control sample with no zymosan-reporter particles. **(C)** Overlay histogram demonstrating the shift in fluorescence of cells with zymosan reporter particles due to oxidation after 10 min (red), 30 min (orange), and 60 min (blue) compared with cells without zymosan-reporter particles (green) for both neutrophils and monocytes.

We used whole blood from four healthy HIV-negative controls to measure phagocytosis and superoxide burst of phagocytes *ex vivo* using zymosan-reporter particles. We first sought to determine the kinetics of zymosan uptake by whole blood phagocytes. The flow cytometry gating strategy to identify neutrophils and monocytes is outlined in Figure 1. Cells that had phagocytosed zymosan-reporter particles were identified and quantified through measurement of the calibration fluor, Alexa Fluor 405.

**Figure 2:**
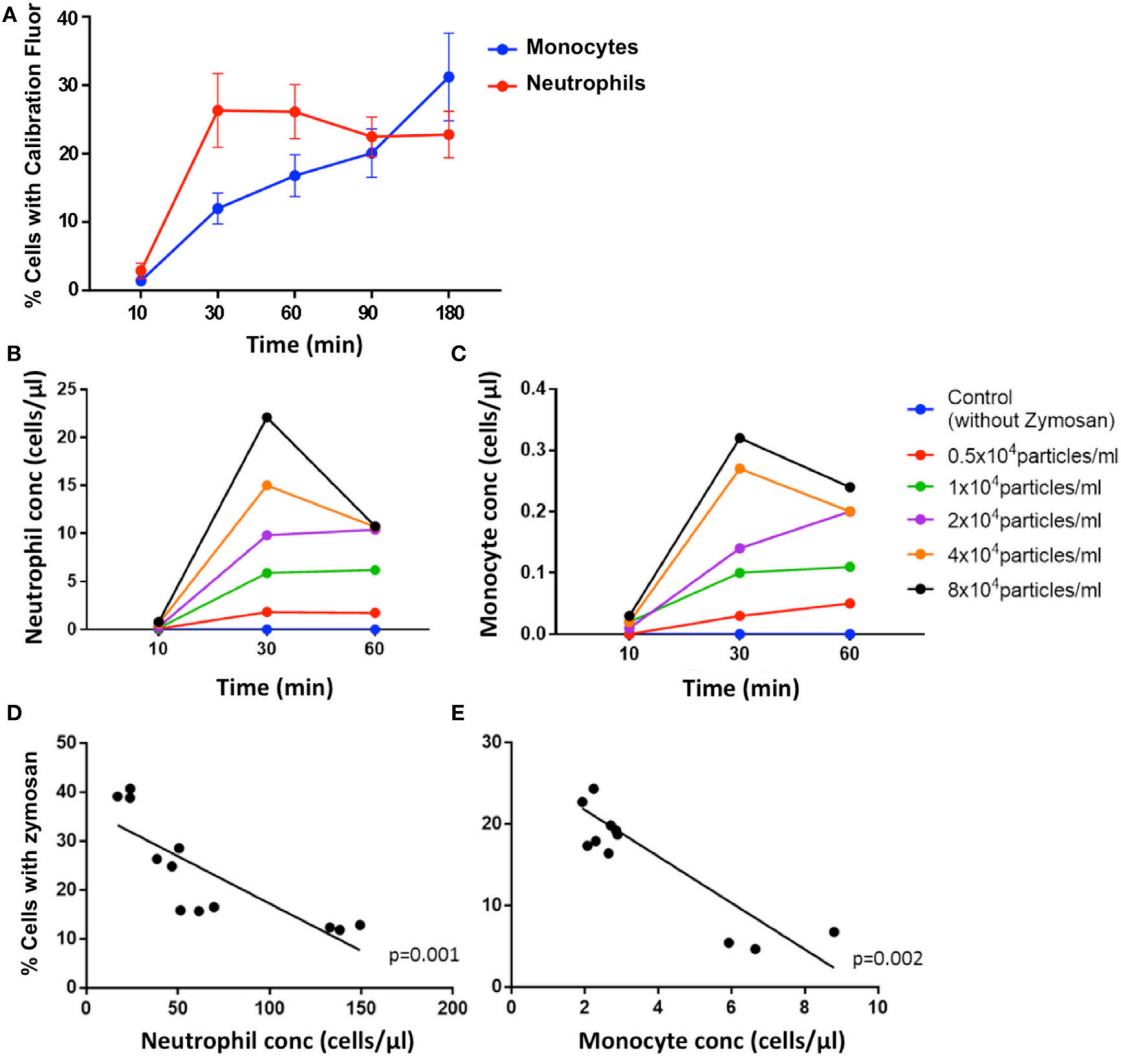
Phagocytosis of zymosan reporter particles by monocytes and neutrophils. **(A)** Proportion of cells that have phagocytosed zymosan over time using blood from healthy individuals incubated with approximately 2 × 10^4^ zymosan particles/ml. Data represent mean values, and the error bars indicate SEM. Concentration of neutrophils **(B)** and monocytes **(C)** that have internalized zymosan reporter particles over time, with varying concentrations of particles. Relationship between the proportion of cells internalizing zymosan reporter particles (at an approximate concentration of 2 × 10^4^ zymosan particles/ml) and absolute cell concentration for neutrophils **(D)** and monocytes **(E)** after 60 min. Each data point represents one sample from each individual done in triplicate. Four healthy volunteers in triplicate are shown in panels **(A,D,E)**, and one health volunteer is shown in panels **(B,C)**.

Zymosan particles were avidly internalized by both neutrophils and monocytes in blood from healthy controls. Uptake was rapid, with a mean of 26% of neutrophils phagocytosing the particles compared with 12% of monocytes by 30 min (Figure 2A). The proportion of neutrophils phagocytosing zymosan did not increase substantially between 30 and 180 min, whereas the percentage of monocytes associated with zymosan-reporter particles increased gradually during the assay. This pattern of uptake was consistent across all healthy controls.

The uptake of zymosan reporter particles by both monocytes and neutrophils is dose dependent as shown in the dose–response curve generated for 0.5 × 10^4^–8 × 10^4^ zymosan particles/ml (Figure 2B). The abundance of the phagocytic cells in whole blood also influences the overall proportion of cells phagocytosing zymosan particles (Figure 2C). The higher the concentration of cells, the lower the proportion of cells carrying the zymosan-reporter signal, shown for both neutrophils and monocytes (Figures 2D,E). This relationship persists throughout the assay and demonstrates the importance of the phagocyte to particle ratio in the kinetics of phagocytosis. Relying solely on internalization of particles to assess phagocytic function is a potential limitation of the assay, as the magnitude of phagocytosis may be influenced by a function of cell concentration and/or cell to particle ratio, rather than cellular deficiencies in phagocytic capacity.

**Figure 3:**
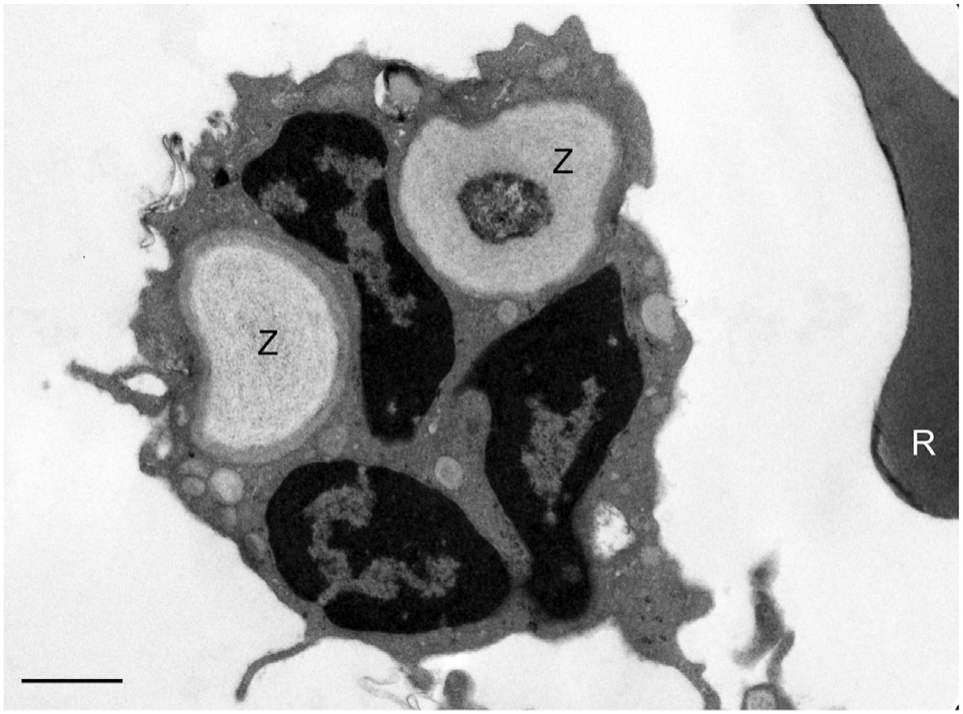
Assessment of phagocytosis of zymosan reporter particles by electron microscopy. An electron micrograph illustrating zymosan particles (Z) inside a neutrophil 60 min post incubation of the reporter particles with whole blood. A red blood cell (R) can be seen to the right of the neutrophil. This image is representative and indicates that the zymosan particles are effectively internalized by cells in suspension. The scale bar = 1 µm.

Electron microscopy of white blood cells from a healthy control whole blood incubated with zymosan particles demonstrates phagocytosis of zymosan particles by peripheral blood phagocytes (Figure 3). The EM images support the assumption that the zymosan reporter signal detected by flow cytometry originates from phagocytosis rather than the association of zymosan particles with the phagocyte surface. Almost without exception, the zymosan particles were observed inside the phagocyte.

### Cell Loss Associated With Zymosan

**Figure 4:**
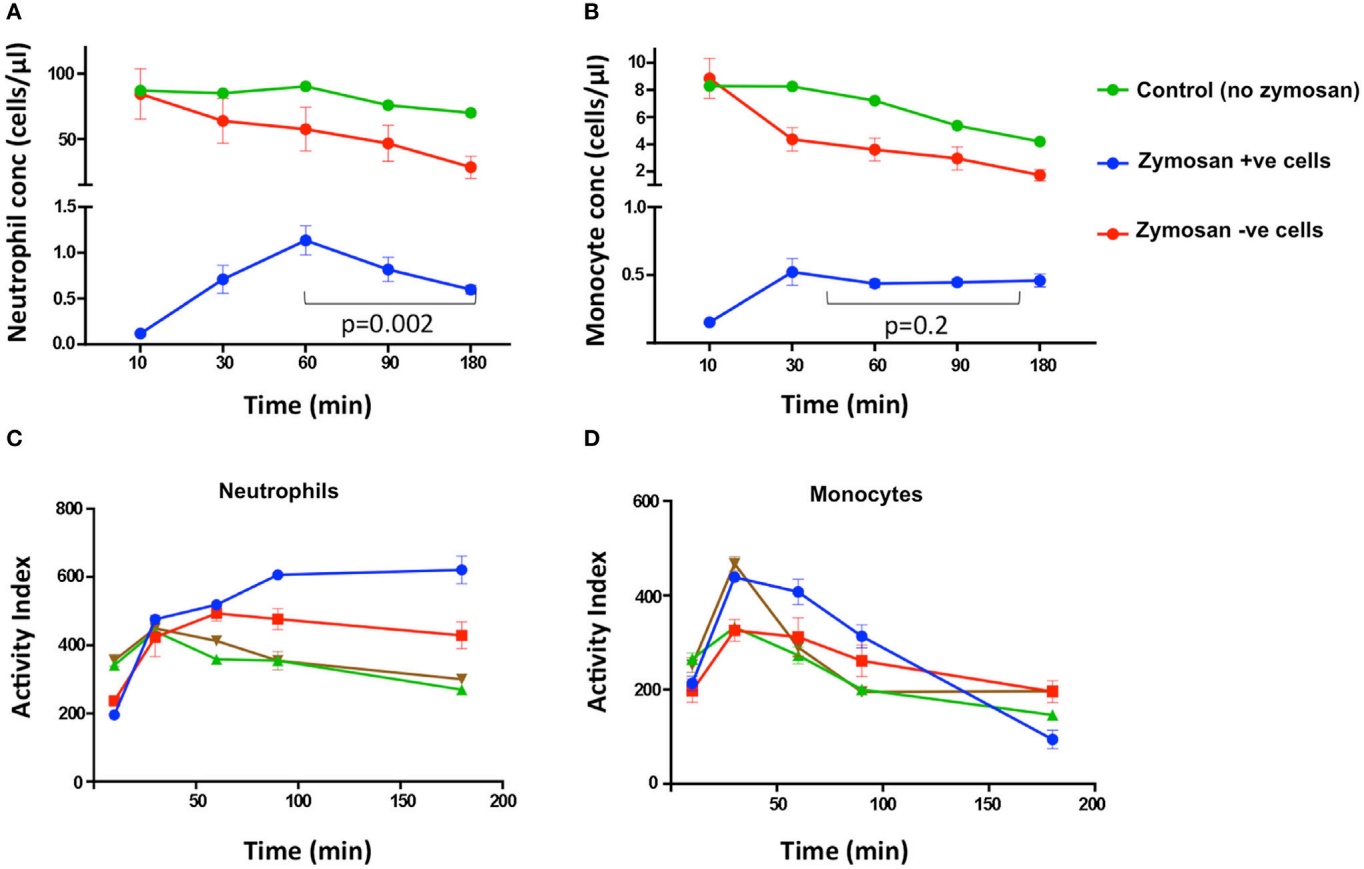
Cell concentrations and activity index (AI) of superoxide burst over time. The concentration of neutrophils **(A)** and monocytes **(B)** at different time points from HIV-negative control samples without zymosan (green), and samples incubated with zymosan reporter beads that had internalized zymosan (blue) or did not internalize zymosan (red). In **(C)** neutrophils and **(D)** monocytes, each color represents the superoxide AI data from different individuals. The AIs are from four healthy, HIV-negative individuals, each performed in triplicate.

To examine the effect of the zymosan particles on cell loss, we compared the samples containing zymosan reporter particles and control samples from the same healthy individuals. The mean concentration of neutrophils and monocytes declined during the assay more rapidly in the presence of zymosan than in control samples, with the largest decline occurring between 90 and 180 min (Figures 4A,B).

Furthermore, the concentration of neutrophils associated with zymosan-reporter signal peaked at 60 min, followed by a decline (Figure 4A). By contrast, the concentration of zymosan-associated monocytes plateaus at 30 min (Figure 4B). However, in both cell types the peak in zymosan uptake coincided with cell loss, suggesting that zymosan plays a role in inducing cell death. This is also supported by increased cell loss at higher concentrations of zymosan in the assay (Figure 2B).

These observations are consistent with neutrophil and monocyte biology. Neutrophils are known to have a short half-life *in vitro*, estimated to be 6–12 h, and do not proliferate (24). Programmed cell death of neutrophils occurs rapidly following phagocytosis of inflammatory particles, and reactive oxygen species may be important triggers for induction of apoptosis (20). In neutrophils that have not phagocytosed zymosan particles, activation *via* direct binding of zymosan to TLR2 and TLR6 or in response to inflammatory cytokine and chemokine production may also contribute to cell death (12). By contrast, monocytes have an estimated half-life of <20 h *in vivo*, although this may be shorter *ex vivo* (25). Monocytes also undergo programmed cell death, unless they migrate to tissues and undergo differentiation into tissue macrophages (26). However, in contrast to neutrophils, inflammatory cytokine production and stimulation *via* TLR2 can promote survival by blocking programmed cell death (27). This may explain why monocytes that had phagocytosed zymosan reporter particles did not substantially decrease in number during the assay.

### Phagocytosis and Superoxide Burst

Superoxide burst activity was measured at 10, 30, 60, 90, and 180 min by comparing fluorescence of cells that had internalized zymosan reporter particles (calibration fluor-positive cells) with the cell population without zymosan (calibration fluor-negative cells) through measurement of the OxyBURST, superoxide sensor signal. The proportion of cells and intensity of superoxide reporter fluorescence increased over the time course of the assay in both monocytes and neutrophils (Figures 1B,C). The intensity of the calibration fluorochrome signal did not increase over time, suggesting the increase in superoxide reporter signal was not due to cells internalizing greater numbers of zymosan-reporter particles but was specific to the oxidase activity (Figure 1B).

Both peripheral blood monocytes and neutrophils showed rapid oxidation within 30–60 min (Figures 4C,D). The kinetics of oxidation in neutrophils and monocytes were similar to macrophages in other studies, with rapid oxidation before an equilibrium being reached, which likely represents cessation of NADPH oxidase activity (6, 15).

When the concentration of zymosan reporter particles was varied, the AI remained constant despite the concentration and proportion of cells taking up zymosan changing. This indicates the assay is able to measure physiological changes in the intensity and duration of phagocytosis and superoxide burst within the phagosome at an individual cell level. This is a significant advance over existing assays, which measure the extracellular accumulation of products of oxidation that is dependent on the summation of phagocytosis and superoxide burst (28). Moreover, because this assay has cellular resolution, the relative contribution of the different phagocyte subsets can be accurately measured. We have also demonstrated that the assay is reproducible with minimal intraindividual variation.

Assays using OxyBURST coupled to IgG coated beads have previously been used to investigate oxidation within macrophage phagosomes (6, 15), and more recently in whole blood (29). The current assay exploiting zymosan as a reporter particle is an important addition to the range of phagocyte functional assays and offers considerable practical and technical advantages in the functional interrogation of whole blood direct from human subjects of interest.

### Assessment of Whole Blood Phagocyte Function in Patients with HIV/TB Coinfection

**Figure 5:**
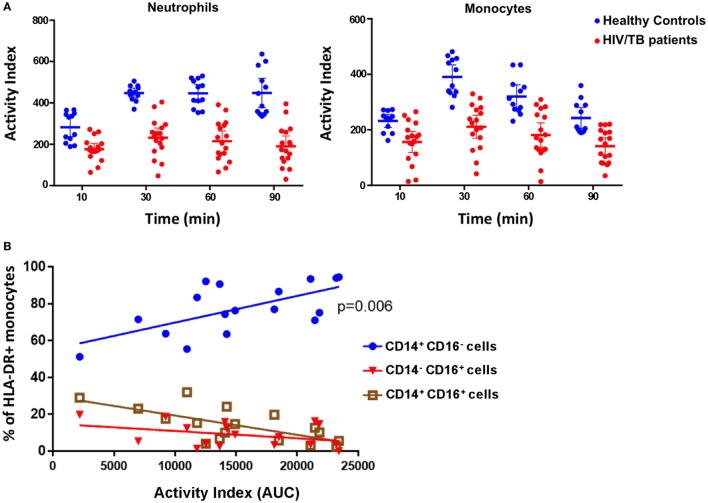
Phagocytosis, superoxide burst, and monocyte phenotypes in peripheral blood phagocytes of HIV/tuberculosis (TB)-coinfected patients. **(A)** The superoxide burst activity index (AI) over time comparing cells from healthy individuals (blue) with cells from HIV/TB-coinfected patients (red). Lines represent means, and error bars are 95% CI. Mean AIs were lower in the HIV/TB patients at all time points analyzed (paired *t*-tests, all *p* < 0.0001). **(B)** The association between the proportion of HLA-DR^+^ monocytes that are CD14^++^CD16^−^ (classical), CD14^+^CD16^+^ (intermediate), and CD14^−^CD16^+^ (non-classical), and monocyte phagocytosis and superoxide burst AI (measured as “area under the curve”). There is a strong association between higher proportions of classical monocytes and increased AI (*p* = 0.006) (*n* = 18). The data in panel **(A)** are from four health volunteers in triplicate, and the data in panel **(B)** are from 18 HIV/TB patients.

The zymosan reporter assay was performed on blood samples obtained from 18 hospitalized HIV-positive patients with laboratory confirmed TB disease to compare phagocytic and superoxide respiratory burst activity in the phagosome between patients and healthy, HIV-negative, controls. The HIV/TB patients had a mean age of 41.4 years, a median CD4 cell count of 108.5 cells/mm^3^ and 13/18 were taking antiretroviral therapy at the point of hospital admission. The HIV/TB-coinfected patients demonstrated marked variation in phagosomal oxidation activity compared with healthy controls. The kinetics were similar to healthy controls with peak activity occurring at 30 min, although overall mean intensity of superoxide burst was significantly reduced throughout the assay (paired *t*-test, all *p* < 0.0001) (Figure 5A). There was also a strong association between increased monocyte superoxide burst activity and the presence of a higher proportion of “classical” CD14^++^CD16^−^ monocytes (Figure 5B, linear regression coefficient 0.0014, 95% CI 0.0005–0.0024, *p* = 0.006). This association is consistent with the suggestion that classical monocytes are thought to specialize in phagocytosis compared with other monocyte subsets (30). However, the superoxide activity in monocytes was not related to the overall concentration of monocytes in patient’s blood (linear regression slope 0.0002, 95% CI −0.0002 to 0.0005, *p* = 0.19), supporting the contention that the superoxide AI was not simply a function of phagocyte abundance.

These data demonstrate that the whole blood assay with zymosan reporter particles is a robust tool for assessing phagocyte function in a clinical setting. The time required to run the assay once the reporter particles have been made is minimal, with the processing of the sample through to acquisition by flow cytometry taking less than 4 h. We also demonstrated this assay can show marked differences between individuals and groups of patients based on clinical phenotype. It is interesting to note that the reduced superoxide burst in the phagocytes from HIV/TB-coinfected individuals observed in this study is consistent with a recent report of impaired innate immune function of monocytes from HIV/TB-coinfected patient cohort in South Africa (31).

## Concluding Remarks

We present a new method for studying whole blood phagocyte functional capacity *ex vivo*. This technique uses fluorescent-tagged zymosan-reporter particles and whole blood, preserving, at least in part, the physiological *in vivo* conditions. It offers several advantages over standard microbiological killing assays because of its speed and simplicity, and its increased resolution whereby cellular responses such as phagocytic capacity and superoxide burst, can be quantified at the level of the individual cell.

We have demonstrated that the assay can be used to characterize immune function and to detect perturbation of cellular function in patients with severe immunological impairment (in HIV/TB-coinfected individuals). This assay is easily adaptable to standard immunological assays based on cell surface marker expression measured by flow cytometry and has the capacity to provide direct functional readouts of immune cell activities. Previously, we have used inert reporter particles to measure rates of phagosomal acidification, intraphagosomal proteolytic and lipolytic activities, as well as superoxide burst in tissue macrophages in culture. These activities are differentially modulated by immune status and infection (7–9). The use of zymosan as an alternative, biologically active carrier particle for whole blood-based assays brings these complex biological readouts into a clinical setting for functional interrogation of patient-derived samples linked to disease status.

## Ethics Statement

The study was carried out in accordance with the recommendations from the London School of Hygiene & Tropical Medicine Research Ethics Committee and the College of Medicine Research Ethics Committee, Malawi, with written informed consent from all subjects. All subjects gave written informed consent in accordance with the Declaration of Helsinki.

## Author Contributions

All the authors contributed to the analysis and interpretation of data and preparation of the manuscript. All the authors have approved the final article.

## Conflict of Interest Statement

The authors declare that the research was conducted in the absence of any commercial or financial relationships that could be construed as a potential conflict of interest.

## References

[1] Kampmann B Tena GN Mzazi S Young DB Levin M Eley B. Novel human in vitro system for evaluating antimycobacterial vaccines. Infect Immun (2004) 72:6401–7. 10.1128/IAI.72.11.6401 15501770 PMC522995

[2] Pattanapanyasat K Sukapirom K Tachavanich K Kaewmoon S. Flow cytometric quantitation of opsonophagocytosis and intracellular killing of *Candida albicans* using a whole blood microassay. Cytometry A (2007) 71:1027–33. 10.1002/cyto.a.20475 17929293

[3] Wallis RS Palaci M Vinhas S Hise AG Ribeiro FC Landen K A whole blood bactericidal assay for tuberculosis. J Infect Dis (2001) 183:1300–3. 10.1086/319679 11262217

[4] DeForge LE Billeci KL Kramer SM Effect of IFN-γ on the killing of *S. aureus* in human whole blood: assessment of bacterial viability by CFU determination and by a new method using alamarBlue. J Immunol Methods (2000) 245:79–89. 10.1016/S0022-1759(00)00279-9 11042285

[5] Hampton MB Winterbourn CC. Methods for quantifying phagocytosis and bacterial killing by human neutrophils. J Immunol Methods (1999) 232:15–22. 10.1016/S0022-1759(99)00147-7 10618506

[6] Podinovskaia M VanderVen BC Yates RM Glennie S Fullerton D Mwandumba HC Dynamic, quantitative assays of phagosomal function. Curr Protoc Immunol (2014) 102:14.34.1–14. 10.1002/0471142735.im1434s102 PMC3920304 24510516

[7] Russell DG Vanderven BC Glennie S Mwandumba H Heyderman RS. The macrophage marches on its phagosome: dynamic assays of phagosome function. Nat Rev Immunol (2009) 9:594–600. 10.1038/nri2591 19590530 PMC2776640

[8] Yates RM Hermetter A Taylor GA Russell DG. Macrophage activation downregulates the degradative capacity of the phagosome. Traffic (2007) 8:241–50. 10.1111/j.1600-0854.2006.00528.x 17319801

[9] Podinovskaia M Lee W Caldwell S Russell DG. Infection of macrophages with *Mycobacterium tuberculosis* induces global modifications to phagosomal function. Cell Microbiol (2013) 15:843–59. 10.1111/cmi.12092 23253353 PMC3620910

[10] Jambo KC Banda DH Afran L Kankwatira AM Malamba RD Allain TJ Asymptomatic HIV-infected individuals on antiretroviral therapy exhibit impaired lung CD4(+) T-cell responses to mycobacteria. Am J Respir Crit Care Med (2014) 190:938–47. 10.1164/rccm.201405-0864OC 25225948 PMC4299580

[11] Di Carlo FJ Fiore JV On the composition of zymosan. Science (1958) 127:756–7. 10.1126/science.127.3301.756-a 13543326

[12] Underhill DM. Macrophage recognition of zymosan particles. J Endotoxin Res (2003) 9:176–80. 10.1179/096805103125001586 12831459

[13] Crespo MS Alvarez Y Valera I Municio C Hugo E Padrn F Eicosanoids in the innate immune response: TLR and non-TLR routes. Mediators Inflamm (2010) 2010 10.1155/2010/201929 PMC2905620 20689730

[14] Lindena J Burkhardt H Dwenger A. Mechanisms of non-opsonized zymosan-induced and luminol-enhanced chemiluminescence in whole blood and isolated phagocytes. Clin Chem Lab Med (1987) 25:765–78. 10.1515/cclm.1987.25.11.765 3440857

[15] Vanderven BC Yates RM Russell DG. Intraphagosomal measurement of the magnitude and duration of the oxidative burst. Traffic (2009) 10:372–8. 10.1111/j.1600-0854.2009.00877.x.Intraphagosomal 19183302 PMC2736473

[16] Nauseef WM. How human neutrophils kill and degrade microbes: an integrated view. Immunol Rev (2007) 219:88–102. 10.1111/j.1600-065X.2007.00550.x 17850484

[17] Fang FC. Antimicrobial reactive oxygen and nitrogen species: concepts and controversies. Nat Rev Microbiol (2004) 2:820–32. 10.1038/nrmicro1004 15378046

[18] Segal AW The NADPH oxidase and chronic granulomatous disease. Mol Med Today (1996) 2:129–35. 10.1016/1357-4310(96)88723-5 8796870

[19] Koziel H Li X Armstrong MY Richards FF Rose RM. Alveolar macrophages from human immunodeficiency virus-infected persons demonstrate impaired oxidative burst response to *Pneumocystis carinii* in vitro. Am J Respir Cell Mol Biol (2000) 23:452–9. 10.1165/ajrcmb.23.4.4084 11017909

[20] Corleis B Korbel D Wilson R Bylund J Chee R Schaible UE. Escape of *Mycobacterium tuberculosis* from oxidative killing by neutrophils. Cell Microbiol (2012) 14:1109–21. 10.1111/j.1462-5822.2012.01783.x 22405091

[21] Gupta-Wright A Fielding KL van Oosterhout JJ Wilson DK Corbett EL Flach C Rapid urine-based screening for tuberculosis to reduce AIDS-related mortality in hospitalized patients in Africa (the STAMP trial): study protocol for a randomised controlled trial. BMC Infect Dis (2016) 16:501. 10.1186/s12879-016-1837-z 27659507 PMC5034586

[22] Rohde KH Veiga DFT Caldwell S Balázsi G Russell DG. Linking the transcriptional profiles and the physiological states of *Mycobacterium tuberculosis* during an extended intracellular infection. PLoS Pathog (2012) 8:e1002769. 10.1371/journal.ppat.1002769 22737072 PMC3380936

[23] Maecker HT Frey T Nomura LE Trotter J Selecting fluorochrome conjugates for maximum sensitivity. Cytometry A (2004) 62:169–73. 10.1002/cyto.a.20092 15536642

[24] Summers C Rankin SM Condliffe AM Singh N Peters AM Chilvers ER Neutrophil kinetics in health and disease. Trends Immunol (2010) 31:318–24. 10.1016/j.it.2010.05.006 20620114 PMC2930213

[25] van Furth R Cohn ZA The origin and kinetics of mononuclear phagocytes. J Exp Med (1968) 128:415–35. 10.1084/jem.128.3.415 5666958 PMC2138527

[26] Fahy RJ Doseff AI Wewers MD. Spontaneous human monocyte apoptosis utilizes a caspase-3-dependent pathway that is blocked by endotoxin and is independent of caspase-1. J Immunol (1999) 163:1755–62. 10438906

[27] Mangan DF Wahl SM. Differential regulation of human monocyte programmed cell death (apoptosis) by chemotactic factors and pro-inflammatory cytokines. J Immunol (1991) 147:3408–12. 1940344

[28] Seitz PM Cooper R Gatto GJ Ramon F Sweitzer TD Johns DG Development of a high-throughput cell-based assay for superoxide production in HL-60 cells. J Biomol Screen (2010) 15:388–97. 10.1177/1087057109359687 20228280

[29] Morton B Mitsi E Pennington SH Reiné J Wright AD Parker R Augmented passive immunotherapy with P4 peptide improves phagocyte activity in severe sepsis. Shock (2016) 46:635–41. 10.1097/SHK.0000000000000715 27488089

[30] Wong KL Yeap WH Tai JJY Ong SM Dang TM Wong SC. The three human monocyte subsets: implications for health and disease. Immunol Res (2012) 53:41–57. 10.1007/s12026-012-8297-3 22430559

[31] Janssen S Schutz C Ward A Nemes E Wilkinson KA Scriven J Mortality in severe human immunodeficiency virus-tuberculosis associates with innate immune activation and dysfunction of monocytes. Clin Infect Dis (2017) 65:73–82. 10.1093/cid/cix254 PMC5849097 28369200

